# Assessing the efficacy of androgen receptor and Sox10 as independent markers of the triple-negative breast cancer subtype by transcriptome profiling

**DOI:** 10.18632/oncotarget.26072

**Published:** 2018-09-07

**Authors:** Khalid N. Al-Zahrani, David P. Cook, Barbara C. Vanderhyden, Luc A. Sabourin

**Affiliations:** ^1^ Ottawa Hospital Research Institute, Centre for Cancer Therapeutics, Ottawa, Ontario, Canada; ^2^ Department of Cellular and Molecular Medicine, University of Ottawa, Ottawa, Ontario, Canada

**Keywords:** androgen receptor, Sox10, bioinformatics, biomarkers, breast cancer

## Abstract

The Androgen Receptor (AR) has recently garnered a lot of attention as a potential biomarker and therapeutic target in hormone-dependent cancers, including breast cancer. However, several inconsistencies exist within the literature as to which subtypes of breast cancer express AR or whether it can be used to define its own unique subtype. Here, we analyze 1246 invasive breast cancer samples from the Cancer Genome Atlas and show that human breast cancers that have been subtyped based on their HER2, ESR1, or PGR expression contain four clusters of genes that are differentially expressed across all subtypes. We demonstrate that Sox10 is highly expressed in approximately one-third of all HER2/ESR1/PGR-low tumors and is a candidate biomarker of the triple-negative subtype. Although AR expression is acquired in many breast cancer cases, its expression could not define a unique subtype. Despite several reports stating that AR expression is acquired in HER2/ESR1/PGR triple-negative cancers, here we show that a low percentage of these cancers express AR (~20%). In contrast, AR is highly expressed in HER2-positive or ESR1/PGR-positive cancers (> 95%). Although AR expression cannot be used as an independent subtype biomarker, our analysis shows that routine evaluation of AR expression in tumors which express HER2, ESR1 and/or PGR may identify a unique subset of tumors which would benefit from anti-androgen based therapies.

## INTRODUCTION

Breast cancer is the most common cancer in women, accounting for approximately 25% of all reported carcinomas worldwide [1]. However, the molecular basis for the various breast cancer subtypes is not fully understood and the vast heterogeneity that exists within each tumor makes treatment an even more difficult task.

Breast cancer subtypes are most commonly classified based on the expression of the Estrogen Receptor (ESR1), Progesterone Receptor (PGR), and/or the Human Epidermal Growth Factor Receptor-2 (HER2) [2–5]. ESR1 and PGR-positive breast cancers make up the Luminal A (Ki-67-negative) and Luminal B (Ki-67-positive) subtypes [2–5]. The Luminal A and B subtypes are the most common, accounting for approximately 65% of all breast cancer cases [4]. The Luminal subtypes tend to have the best prognostic outcome of all subtypes and these tumors generally respond well to hormone therapy [6]. The HER2-positive subtype is characterized by overexpression and amplification of HER2 with a prevalence of approximately 25% and a poor prognosis due to its association with highly metastatic breast cancers [4, 7–9]. The monoclonal antibody, Trastuzumab (Herceptin), in combination with chemotherapy is currently the best form of treatment for HER2-positive breast cancers [10]. Lastly, the triple-negative breast cancers (TNBC) are defined by those that do not express HER2, ESR1, or PGR [5]. TNBCs account for approximately 10% of all cases and have the worst prognosis and survival rates, as major therapeutic targets have not yet been identified in this subtype [4]. As biomarkers and/or drivers have not been fully identified for this subtype, chemotherapy remains the standard of care treatment for systemic TNBC [11–13].

The Androgen Receptor (AR) is a steroid hormone nuclear receptor that is regulated through cytoplasmic binding of testosterone or dihydrotestosterone and subsequent translocation to the nucleus and activation of gene transcription [14]. In addition to its well characterized role in sexual development [15], AR has gained increasing attention as an important mediator of hormone-dependent cancers and a novel therapeutic target in breast cancer [16–18]. Increased expression, nuclear localization and/or phosphorylation of AR have now been observed in a few various breast cancer subtypes [17, 19, 20]. Several studies and systematic meta-analyses have now demonstrated that higher levels of AR are associated with a better prognostic outcome and a reduction in metastatic burden [21–23]. Our lab has previously identified that AR expression is acquired in a murine model of HER2-positive breast cancer following Periostin deletion [20]. We reported that these tumors were of a molecular apocrine histology and have since gained an interest in understanding how AR could be used as a possible biomarker of a specific subtype of breast cancer.

There is current controversy in the literature as to whether AR expression can be used as a biomarker for HER2-positive, ESR1-positive or TNBC, with much of the literature reporting the highest expression in TNBC tumors [21, 24–28]. Most reports indicating that AR is overexpressed in breast cancers are concluded based on immunohistochemical staining. A recent report, which included a review of 23 other TNBC studies, reported that a high number of TNBC tumors acquire AR expression [26]. However, nine of these studies used a very low threshold of 1% positive cells to define acquired AR expression, with eleven others using 10% positive cells as the cut-off. Although the methods for detecting ESR1 or HER2 in breast cancer biopsies by immunohistochemistry are well established [29], AR immunohistochemistry performed on tumor biopsies is not routine or standardized leading to a wide variation in the reported number of AR-positive breast cancer cases [27, 28]. Given this large degree of variability, more consistent methods for determining AR status in the clinic are required.

To assess the subtype distribution of AR expression across human breast cancers, we interrogated The Cancer Genome Atlas containing RNA-Seq data from 1246 invasive human breast cancer samples. Using this dataset, we have shown that AR is highly expressed in over 95% of HER2-, ESR1- or PGR-positive tumors while TNBCs tend to express AR less frequently (~20%).

## RESULTS

### Luminal A/B, HER2-positive and triple-negative subtypes can be defined by four distinct gene expression signatures

**Figure 1:**
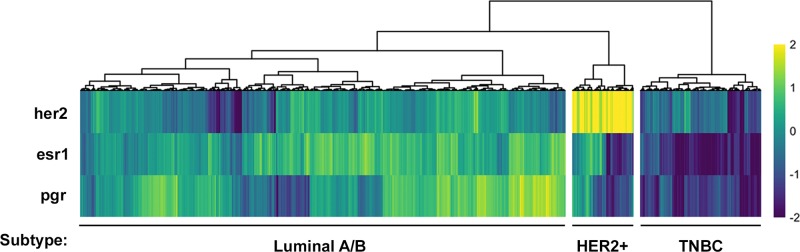
Stratification of human breast cancer samples from *The Cancer Genome Atlas* database into three putative breast cancer subtypes Receptor heatmap of HER2, ESR1 and PGR expression (z-score of log2 counts) across all samples. Each column represents the expression values for an individual patient sample. Hierarchical clustering was used to group patients by the expression patterns of each receptor. This clustering revealed a group of putative TNBC, HER2+ and Luminal A/B patients.

We first aimed to stratify the RNA-Seq data available from The Cancer Genome Atlas according to the three major breast cancer subtypes: HER2-positive, ESR1/PGR-positive, and triple negative. Gene-level quantifications from RNA-Seq data of 1246 invasive breast cancer samples from The Cancer Genome Atlas [30] were clustered based on their expression of HER2, ESR1, and PGR. This resulted in three distinct putative subtypes: a HER2-positive, a Luminal A/B subtype which is ESR1/PGR-positive, and a TNBC subtype lacking expression of all three receptors (Figure 1).

**Figure 2:**
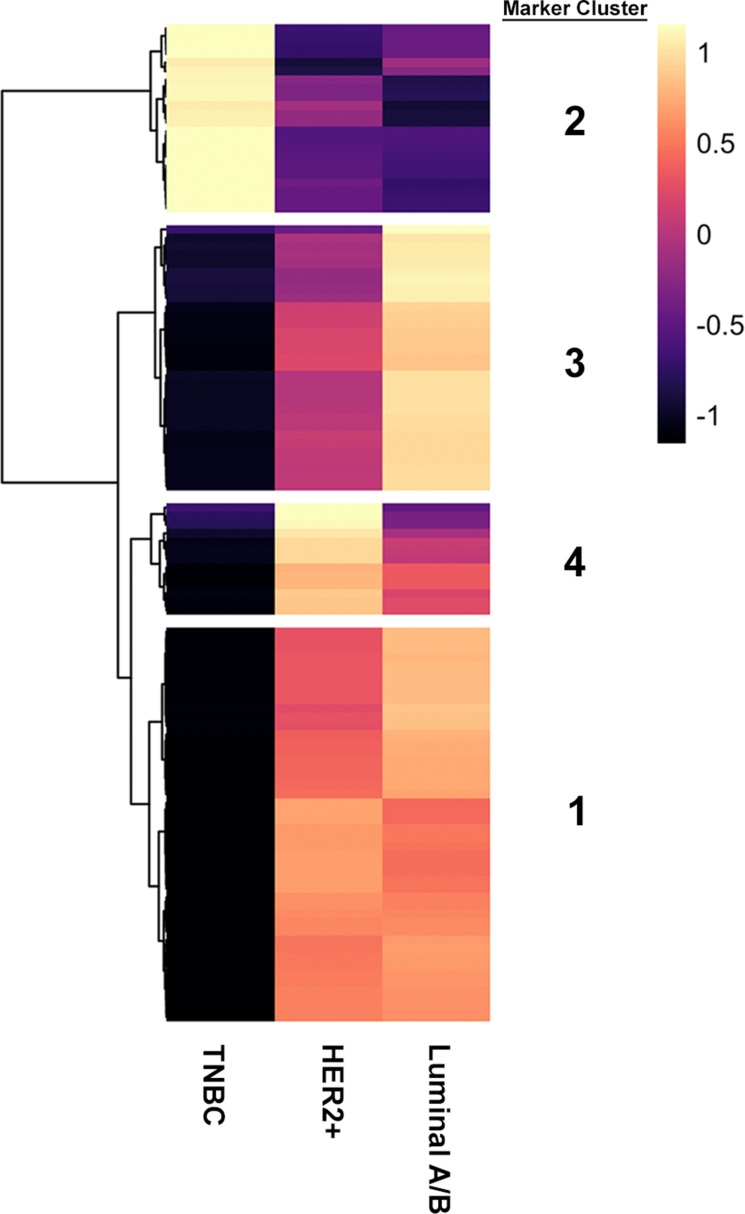
Four distinct marker clusters can be stratified from three putative breast cancer subtypes Marker heatmap showing the average expression (z-score) of 112 marker genes across the three molecular subgroups identified in panel A. Each row represents an individual gene. Marker genes were identified by using an ANOVA to identify genes that are differentially expressed across molecular subtypes. Differentially expressed genes were defined as those with an FDR-adjusted p-value < 0.01 and a log2 fold-change > 4 between the lowest and highest expressing subtype. Hierarchical clustering was used to define four groups of marker genes with similar expression patterns across subtypes.

Next, we sought to define an expanded gene expression signature associated with these subtypes to identify novel biomarkers. Additionally, we were interested in assessing whether AR could be used to define its own unique subtype. To perform this, we identified differentially expressed genes (ANOVA, FDR < 0.01) with a minimum log2 fold change of 4 (16-fold) across subtypes. This resulted in four clusters of genes with distinct expression patterns between subtypes, which we have termed “Marker Clusters”, comprising 112 genes (Figure 2; Table 1).

**Table 1:** Top 20 identified genes for each Marker Cluster identified across HER2+, Luminal A/B and TNBC subtypes

Marker Cluster 1 (Mosaic)	Marker Cluster 2 (High in TNBC)	Marker Cluster 3 (Luminal A/B)	Marker Cluster 4 (HER2+)
AR	SOX10	PGR	ERBB2
PIP	ROPN1	ESR1	CEACAM5
CLC7A2	GABRP	CCDC170	CEACAM6
ABCC8	VGLL1	GRPR	ABCC11
F7	MSLN	WNK4	NXPH1
BCAS1	CA9	CHAD	ABCC12
CA12	GABBR2	AFF3	GRB7
BPIFB2	HORMAD1	GFRA1	PNMT
DHRS2	ZIC1	CPB1	MUCL1
TMC5	ART3	CLSTN2	LRRC26
ARG2	FABP7	PGLYRP2	UGT2B11
GATA3	A2ML1	KCNJ3	PP14571
SCGB2A2	FDCSP	NEK10	DSCAM-AS1
LRRC31	PRAME	GRIK3	
MLPH	KRT16	DNALI1	
FOXA1	SBSN	SERPINA6	
HMGCS2	PPP1R14C	SYT9	
TTC6	CT83	NAT1	
CYP4B1	NKX1-2	CST9	
SYTL5		AGR3	

The four Marker Clusters (and the putative group that they define) are provided with the top genes identified for each cluster with an FDR-adjusted p-value <0.01 and a log2 fold-change > 4 between the lowest and highest expressing subtypes. For clusters with more than 20 genes, only the top 20 are shown here. These genes correspond to those identified by hierarchical clustering in Figure 2.

### Luminal A/B and HER2-positive breast cancers have a defined gene signature

**Figure 3:**
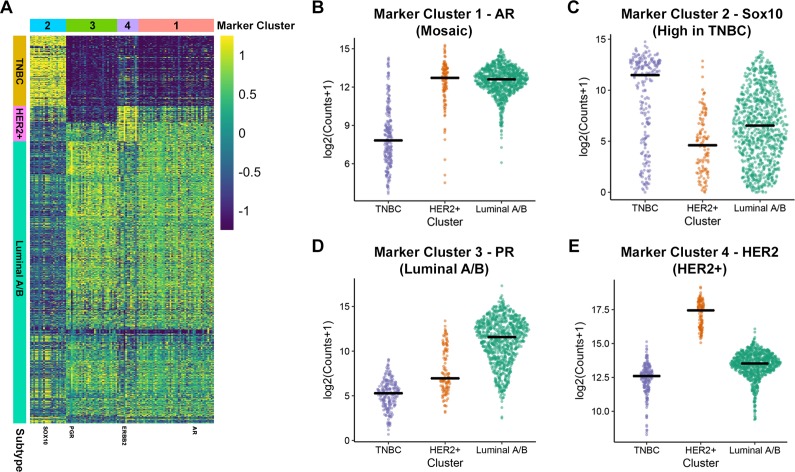
Identification of gene markers for HER2+, Luminal A/B and TNBC **(A)** Marker heatmap showing the expression (z-score of log2 counts) of 112 marker genes. Each row represents the expression of a single gene across each patient sample (columns). The rows of the heatmap are ordered identically to the receptor heatmap in Figure 1. **(B-E)** Expression of a representative identified marker across molecular subtypes is shown for each of the four identified marker groups. Each plot provides the distribution of log2 expression values of AR (B), SOX10 (C), PGR (D) and HER2 (E) across subtypes. The black line represents the median expression value for each subtype.

The Marker Cluster 3 corresponded to genes whose expression is highest in the Luminal A/B subtype and contained the canonical markers ESR1 and PGR (Figure 3, Table 1). Other genes within this cluster include CCDC170, WNK4 and AGR3 (Table 1) which have been reported to be implicated in these cancers [31–33].

Marker Cluster 4 genes are enriched in HER2-positive breast cancers and includes ERBB2 (HER2), GRB7 and CEACAM6 (Figure 3, Table 1). This subtype was well-defined, with all samples expressing higher levels of HER2 than the mean of all samples. As with the Luminal cluster of genes, most genes making up this cluster have been heavily implicated in HER2-positive breast cancer [34, 35].

### Two marker clusters define a TNBC gene signature

Two remaining Marker Clusters corresponded to gene expression patterns that define the TNBC subtype. Firstly, we identified Marker Cluster 1, termed the “mosaic” cluster, as the grouping of genes that were significantly increased in both the HER2-positive and Luminal A/B subtypes when compared to the TNBC subtype (Figure 3, Table 1). Therefore, this cluster represents genes whose RNA expression values are lowest in the TNBC subtype. Interestingly, this cluster contains AR and one of its target genes, PIP [36] which are both expressed at much lower levels in TNBC tumors when compared to the other two subtypes (Figure 3). Further investigation into AR expression reveals that only two major isoforms are expressed in human breast cancers, with both isoforms showing a significantly lower level of expression in the TNBC subtype compared to both other subtypes (Supplementary Figure 1). Additionally, HER2-positive and Luminal A/B tumors express that same isoforms of AR suggesting a similar function for AR in these subtypes.

Given that the “mosaic” Marker Cluster contains genes that are generally downregulated in TNBC tumors compared to HER2-positive and Luminal A/B tumors, we investigated Marker Cluster 2, which contained genes that are significantly enriched in the TNBC subtype. The most striking differences in our differential expression analysis were the genes that show increased expression in the absence of HER2, ESR1 and PGR. This can be observed in the first column of the marker heatmap in Figure 2 as well as the top rows of marker heatmap averaged across clusters in Figure 3A. SOX10 is among the most differentially expressed genes within the TNBC subtype (Figure 3C) and has gained increasing attention as a novel biomarker for these tumors over the past decade [37, 38]. Within the TNBC cluster, tumors tend to express higher levels of SOX10 than any other subtype (Figure 3C), providing further evidence that SOX10 is in fact a biomarker of TNBC. This is strikingly similar to the 73.3% of Luminal A/B tumors with PGR expression – a bona fide Luminal A/B marker – greater than its mean across all samples.

### Sox10 can be used as an independent biomarker of the TNBC subtype

**Figure 4:**
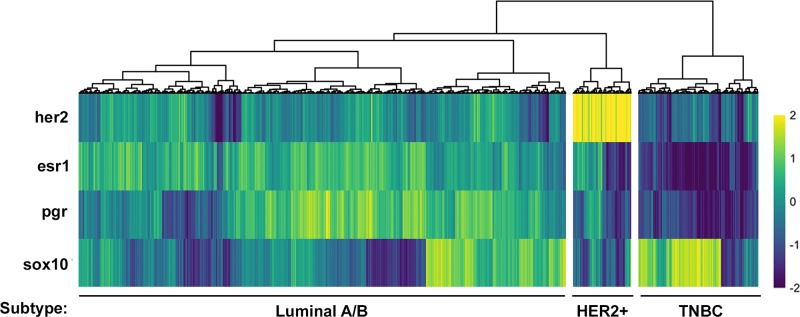
SOX10 expression can be used to independently define a TNBC subtype Heatmap of HER2, ESR1, PGR and SOX10 expression (z-score of log2 counts) across all samples. Each column represents the expression values for an individual patient sample. Hierarchical clustering was used to group patients by the expression patterns of each gene. This clustering revealed a group of putative TNBC, HER2+ and Luminal A/B patients which was almost identical to that in Figure 1 suggesting that SOX10 expression defines a TNBC subtype.

The current strategy for identifying the TNBC subtype is to assess patients for the absence of HER2, ESR1 and PGR, which is the approach that we used when first analyzing the TCGA dataset (Figure 1). However, identifying a gene or set of genes whose expression defines a subtype could improve clinical diagnosis, may provide a better insight into the disease and allow for the identification of novel therapeutics. One of the strongest candidates for a bona fide biomarker of the TNBC subtype that we have identified is Sox10 (Figure 3A, 3C; Table 1). To further validate whether Sox10 is a biomarker of the TNBC subtype, we performed unsupervised clustering of the TCGA dataset based on the expression of HER2, ESR1, PGR and SOX10. We predicted that if Sox10 was a biomarker of the TNBC subtype, adding its expression analysis to the clustering we performed with HER2/ESR1/PGR alone (Figure 1) would not change the proportion of patient samples within each putative subtype. Confirming this hypothesis, the three putative subtypes remained effectively identical when SOX10 expression was added to the clustering analysis (Figure 4). Although SOX10 expression is highest in the TNBC subtype, it is interesting to note that most HER2-positive samples have very low expression of Sox10 while the Luminal A/B subtype has more variable SOX10 expression (Figure 4).

### Sox10 correlates with a more basal/stem-like phenotype in the TNBC but not luminal A/B subtype

**Figure 5:**
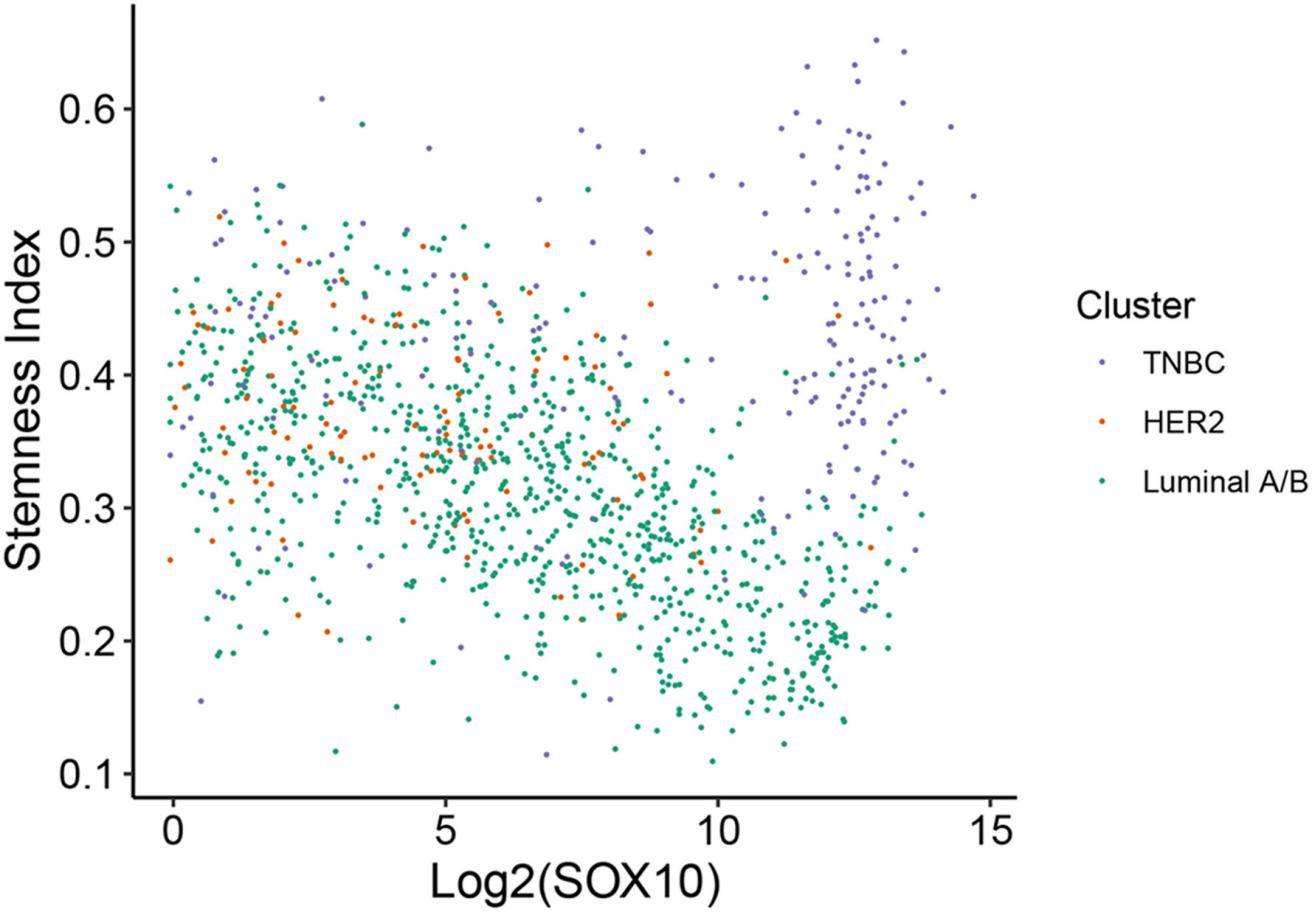
SOX10 expression correlates with a more basal/stem-like phenotype in TNBC Stemness index for all 1246 patients from the TCGA dataset was previously calculated [42] and plotted against SOX10 expression (log2). Individual samples were color coded according to their identified molecular subtype (Figure 1).

The TNBC subtype is often associated with increased stem cell activity which may result in these tumors being more resistant to conventional drug therapies [11, 39]. We have also identified that SOX10 is amongst the most differentially upregulated genes in the TNBC subtype (Table 1). Interestingly, SOX10 transcriptional activity has been shown to be sufficient to reprogram pluripotent cells into a multipotent state and more specifically has been shown to regulate the stem/progenitor activity of mammary epithelial cells [40, 41]. Therefore, we aimed to assess whether SOX10 expression was correlated with an increase in stemness. To perform this analysis, we utilized a recent study in which the stemness indices for all TCGA samples were calculated [42] and plotted stemness index against SOX10 expression (Figure 5). Interestingly, high SOX10 expression correlated with high stemness only in the TNBC subtype, whereas Luminal A/B tumors which had higher levels of SOX10 tended towards a lower stemness index (Figure 5). These data suggest that SOX10 alone is not the only driver of the stem-like phenotype or that Luminal A/B and HER2 tumors possess a pro-differentiation program that is able to overcome the basal/stem-like state driven by SOX10.

### Androgen receptor expression is low in TNBC but acquired in a subset of HER2, ESR1 or PGR-positive tumors

**Figure 6:**
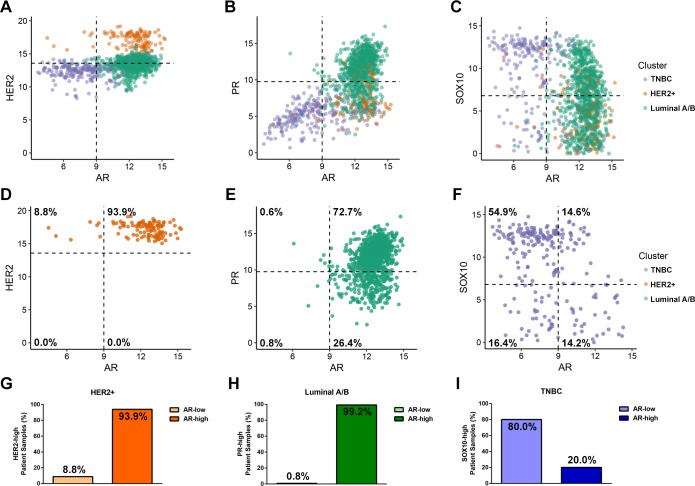
Androgen Receptor is expression is highest in the HER2-positive and Luminal A/B subtypes **(A-C)** Scatter plots showing AR expression (log2) and the expression of markers for each molecular subtype. HER2 was used as a marker of the HER2+ subtype (A), PR for the Luminal A/B subtype (B) and Sox10 for the TNBC subtype (C). Each point represents an individual patient sample and is colour coded according to which subtype that sample stratified with from Figure 1. The dotted lines along the axes is used to visually quadrant the data points. For the subtype markers, this line corresponds to the mean expression value of the indicated gene across all 1246 patient samples, and for AR, the line was manually set at a value that splits the bimodal distribution of AR expression observed across cell types. **(D-F)** Identical plots to (A-C), but reduced to only samples from the HER2-positive (D), Luminal A/B (E) or TNBC (F) subtypes. The percentage of patient samples that fall within each quadrant of expression is provided. **(G-I)** The percentage of AR-low or AR-high expressing tumor samples within the HER2-positive (G), Luminal A/B (H) or TNBC (I) subtypes which express high levels of their corresponding markers (HER2, PGR and SOX10, respectively).

AR expression was observed to be significantly increased in the mosaic Marker Cluster, which was characterized by having higher expression in HER2-positive and Luminal A/B subtypes when compared to TNBCs (Figure 3B). Therefore, we set out to identify the extent of AR expression within all three breast cancer subtypes. To address this, we compared AR expression with the strongest identified marker of each subtype: HER2, PGR or SOX10 (Figure 6A-6F). Consistent with our clustering, HER2 (Figure 6A, 6D), PGR (Figure 6B, 6E) and SOX10 (Figure 6C, 6F) were most expressed in the HER2-positive, Luminal A/B and TNBC subtypes, respectively. Interestingly, AR expression tends to be higher in the HER2- and PGR-positive samples but lower in the SOX10-positive cases as seen by a leftward shift of SOX10-high/AR-low samples (Figure 6C). Together, these data suggest that AR expression is lower in the majority of TNBC samples.

When comparing HER2 and AR expression 93.9% of HER2-positive patients express high levels of both receptors (Figure 6D, 6G). Next, we compared PGR and AR expression within the Luminal A/B subtype. Here, we observed that under 2% of the entire subtype has low expression of AR, with over 98% expressing high levels of AR independent of PGR expression levels (Figure 6E). If we consider only Luminal tumors with high PGR expression, AR is highly expressed in 99.2% of those samples (Figure 6H). Lastly, we compared AR expression with the novel TNBC marker, SOX10. Within this subtype we observe that 71.3% of TNBC tumors display low levels of AR, suggesting that AR independently cannot be considered a biomarker for the TNBC subtype (Figure 6C, 6F, 6I).

**Figure 7:**
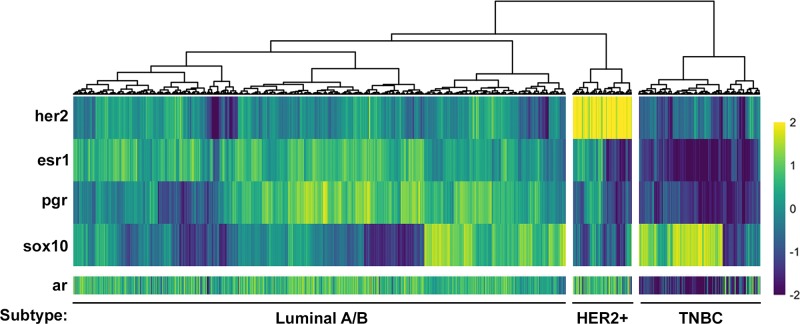
Androgen Receptor expression is inversely correlated with the TNBC marker, Sox10 AR expression data (z-score of log2 counts) was plotted for each patient sample from an unsupervised clustering of the TCGA dataset using HER2, ESR1, PGR and SOX10 (see Figure 4) to define HER2-positive, Luminal A/B and TNBC subtypes. AR expression values are inversely correlated with SOX10 expression and are lowest in the TNBC subgroup.

To provide further evidence for the lack of AR expression in the TNBC subtype, we utilized our hierarchical clustering of patient samples based on HER2, ESR1, PGR and SOX10 and assessed the expression of AR across each patient (Figure 7). The patient samples are ordered identically to Figure 4, with AR expression not affecting the clustering. This confirmed that AR expression is in fact lowest in the SOX10-positive TNBC patient samples, inversely correlated with SOX10 expression (Figure 7). In fact, the few sporadic patients in the TNBC subtype with low SOX10 expression have a higher than average expression of AR (Figure 7).

**Figure 8:**
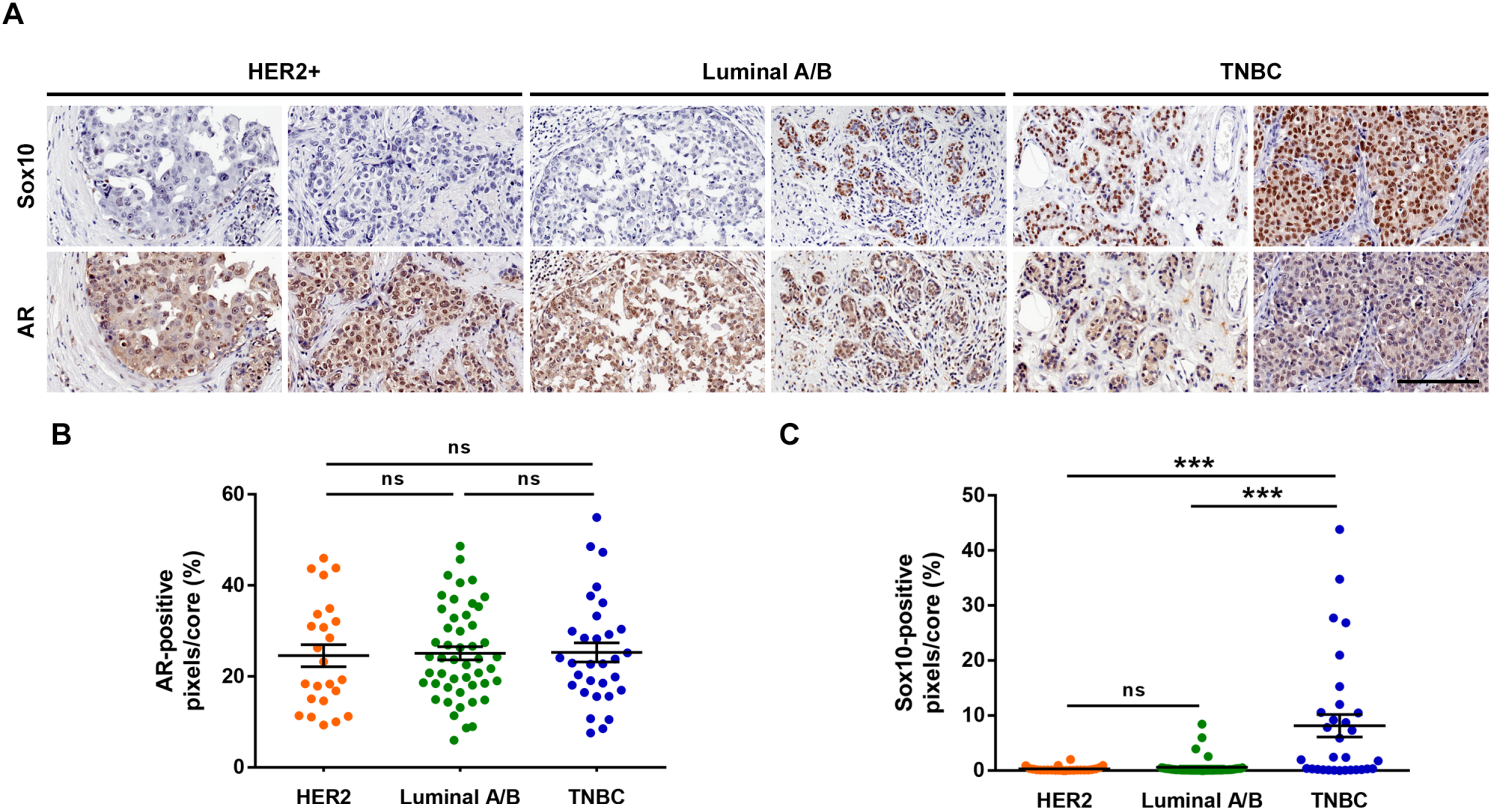
SOX10 histochemistry can be used as a predictor of TNBC **(A)** Human breast cancer TMA BR20810 was purchased from US Biomax. Serial slides containing 104 breast cancer cases were stained for SOX10 and AR. Two representative cores for each subtype are shown. **(B, C)** Each core was quantified for the positive-pixel intensity for both AR (B) and SOX10 (C) staining. No differences were observed between subtypes for AR staining. However, a significant increase in SOX10 staining intensity was observed in the TNBC tumor cores. Scale bar = 200 μm.

To corroborate the findings drawn from our analysis, we performed immunohistochemical staining of a human breast cancer TMA for both SOX10 and AR. In accordance with our previous analysis, Sox10 histochemistry revealed an increased staining intensity and more prominent nuclear localization in the TNBC tumors than within the HER2 and Luminal A/B cores (Figure 8A, 8C). Additionally, quantification of the AR staining showed no significant differences between subtypes, although qualitatively a weaker cytoplasmic signal was noticed within TNBC tumor cores when compared to the other subtypes (Figure 8A, 8B). Therefore, at the level of both the transcriptome and proteome, SOX10 acts a strong marker of the TNBC subtype.

## DISCUSSION

To assess the subtype distribution of AR amongst the three major breast cancer subtypes, we stratified The Cancer Genome Atlas into putative HER2-positive, Luminal A/B and Triple Negative subtype using the available RNA-seq data [30]. Interestingly, molecular subtyping of breast cancers in the clinic is routinely based on immunohistochemical analysis of HER2-, ESR1- and PGR-expression from tumor biopsies and is rarely assessed at a transcriptional level [29]. Consistent with the immunohistological molecular subtyping, our hierarchical clustering of patient samples based on HER2, ESR1 and PGR expression resulted in a similar distribution of subtypes, with most samples falling within the ESR1/PGR-positive luminal subtype and TNBC making up the minority of samples [2–9].

To identify potential biomarkers within each molecular subtype we chose to perform our analysis using a log2 fold change of 4 (16-fold) cut-off. This was chosen as any biomarkers defining these subtypes should have a robust difference in expression. This cut-off resulted in 112 significantly changed genes between each subtype, with no more than 25 genes comprising any one cluster. We believe that these clusters form a manageable list of genes that can be considered as potential biomarkers for each group. Although arbitrarily defined, the identification of HER2 and ESR1/PGR as markers of their corresponding subtype validates our approach. More stringent cut-offs, such as log2 fold change of 5 (32-fold) or greater may be applied to this data set to identify more rigorous biomarkers of each subtype.

Interestingly, our analysis identified distinct gene signatures for both the Luminal A/B and HER2-subtypes. A number of these genes have been validated as either important for the progression of their respective subtype or reported to be a biomarker for that disease [31–35]. One caveat to our approach is that we cannot determine whether the expression of any given gene is downregulated in two of the three subtypes that we defined (e.g. promoter hypermethylation) or, conversely, whether that gene is induced in one subtype (e.g. chromosome duplication). One possible method to address these issues is to include matched normal tissue as a baseline measure of gene expression to elucidate whether expression is gained or lost during tumorigenesis. These gene signatures may be very important in deriving more-thorough subtype classifications beyond the hormone receptors as well as novel therapeutic strategies in the treatment of these cancer subtypes.

One of the most induced genes within the TNBC subtype was SOX10 showing that it may be used as a potential biomarker of the TNBC subtype. This also raises the interesting possibility that SOX10 targeted therapies may be beneficial in treating TNBC patients. Although interfering with transcription factor activity can be challenging therapeutically, it has proven to be successful in a number of clinical trials (reviewed in [43]). Furthermore, identifying specific target genes, pathways and processes regulated by SOX10 in the context of breast cancer may provide a novel therapeutic approach in the treatment of TNBCs.

Although SOX10 is highly expressed in the TNBC cluster, we have also observed that a subset of Luminal A/B tumors also express SOX10 (Figure 3C, 4, 6C). Initially this observation would argue against SOX10 being an independent marker of the TNBC subtype. However, Luminal A/B tumors which have high levels of SOX10 also express high levels of AR. Assessing tumors for both AR and SOX10 may provide a novel method for distinguishing TNBC tumors from the small proportion of Luminal tumors that express SOX10. Therefore, screening Luminal A/B patients for SOX10 may provide a new avenue for treatment for endocrine therapy resistant tumors. Further gene expression analyses could reveal whether these SOX10-positive luminal cases dichotomize the Luminal A and Luminal B patients. Of note, the Luminal A/B patients fall within three major subgroups which can be defined by high ESR1-expression with low PGR-expression, low ESR1-expression with high PGR-expression or a median level of expression of both receptors (Figure 1, 3). Interestingly, high SOX10-expression within the Luminal A/B subtype falls within this last subgroup with an average expression of both ESR1 and PGR.

A dichotomy exists in the current literature as to whether AR can be used as a biomarker or a “fourth receptor” along with HER2, ESR1 and PGR to define the TNBC subtype, with many sources claiming AR to be a TNBC marker [21, 24, 25]. Although many of these studies report that a small percentage of TNBC cases are of a luminal androgen receptor positive (LAR) molecular classification, we believe that caution should be taken in the use of “biomarker” in the context of the whole TNBC subtype as we have shown that SOX10 and AR are often reciprocally expressed. The high degree of correlation between AR and HER2, ESR1 or PGR lends further support to the so-called Quadruple Negative Breast Cancer (QNBC) hypothesis, which may be a stratification of the TNBC subtype with high expression of SOX10 [44]. Although these QNBC tumors would probably not respond to anti-AR based therapeutics, the 20-30% of TNBC cancers that do in fact express AR might and most likely belong to the LAR molecular subgroup [25].

As AR is often co-expressed with HER2 specifically in the HER2-positive subtype, we believe that these patients may also be responsive to anti-androgen based therapies. Currently, Herceptin is the best form of treatment for HER2-positive breast cancers, but often patients develop resistance due to constitutive activation of downstream HER2 signalling [10]. Targeting of AR may have a large impact as a novel therapeutic strategy for treating Herceptin resistant HER2-positive breast cancers or could even be considered as a strong candidate for combination therapy. As with the HER2-positive subtype, the majority of Luminal A/B tumors express high levels of AR. Although these cancers have the best prognostic outcome and respond relatively well to hormone therapy, they may also benefit from anti-androgen based therapies.

AR expression is elevated in samples that have high expression of HER2, ESR1, or PGR. Therefore, it is unlikely that these receptors are all independently responsible for maintaining AR expression. Similarly, this would suggest that it is unlikely that SOX10 is acquired in TNBC tumors due to the loss of AR. We have also corroborated this by immunohistochemical analysis of primary patient tumor cores where we observed no differences in AR expression between HER2, Luminal A/B, or TNBC samples despite the TNBC subtype showing a significant increase in SOX10 expression (Figure 8). Additionally, SOX10 expression did not correlate with increased stemness within the Luminal A/B or HER2-positive subtypes despite having a strong correlation in TNBC tumors (Figure 5), suggesting that these tumors may possess pro-oncogenic signals that bypass the basal/stem-like phenotype of TNBC tumors and keep them in a differentiated state. Understanding the mechanisms of SOX10 induction in breast cancers and the signaling that regulated SOX10 activity is a subject of ongoing research and will shed light on potentially new therapeutic targets for TNBC cancers.

Taken together, our data suggests that AR is expressed in many breast carcinomas, however unlike SOX10, it cannot be used as an independent biomarker. Here, our RNA-seq based approach for profiling the three major breast cancer subtypes matches what is currently reported in the literature with immunohistochemical based profiling. Additionally, by using RNA-seq data we performed a genome-wide exploration for markers associated with each subtype. However, our analysis from this same dataset suggests that AR expression is variable across all subtypes. Although a discordance between the levels of RNA transcripts and protein expression does exist, further analyses are required to determine whether current immunohistochemical approaches can accurately predict AR-positivity in breast cancer. Together, we have shown that AR is not a good biomarker for the existing subtypes of breast cancer, although its expression could be used to define further subtype stratification.

## MATERIALS AND METHODS

### Access of the cancer genome atlas database

Gene-level RNA-seq (v2) counts from the cohort of invasive breast carcinoma samples from The Cancer Genome Atlas (n=1246, [30]) were collected using the R package recount2 [45]. Counts were scaled to account for differences in library size across samples.

### Stratification of molecular breast cancer subtypes

HER2, ESR1 and PGR expression (z-score of log2 counts) was calculated from each of the 1246 invasive breast carcinoma samples. Hierarchical clustering was used to group patients by the expression pattern of each receptor, revealing a group of putative TNBC, HER2-positive and Luminal A/B patients.

### Identification of marker genes

Marker genes were identified using an ANOVA to identify genes that are differentially expressed across all molecular subtypes. Differentially expressed genes were filtered with a Benjamini-Hochberg-adjusted p-value < 0.01 (ANOVA) across all subtypes and a minimum log2 fold change of 4 (16-fold) between the subtype with the lowest expression and the one with the highest expression.

### Immunohistochemistry

Human breast cancer tissue microarrays (BR20810, US Biomax) were deparaffinized and subject to antigen retrieval in 10 mM citrate buffer (pH 6.0) for 10 minutes in a pressure cooker. Endogenous peroxidase was quenched with 3% hydrogen peroxide for 15 minutes. Sections were blocked in 5% goat serum in PBS for an hour and incubated with either Sox10 (NBP2-44474, Novus) or AR (ab74272, abcam) primary antibody overnight at 4°C followed by incubation with the appropriate HRP-conjugated secondary at room temperature for 30 minutes. Antibodies were incubated in blocking solution. Sections were then incubated in DAB substrate (Sigma Aldrich) and counterstained with Haematoxylin. Sections were dehydrated in ethanol prior to clearing in xylene before mounting the slides. Positive pixels were enumerated using an Aperio Scanscope.

### Data analysis scripts

All analysis scripts required to reproduce these findings are available at: https://github.com/dpcook/tcga_breast_cancer.

## SUPPLEMENTARY MATERIALS FIGURES AND TABLES



## Abbreviations

AR: Androgen Receptor
HER2: Human Epidermal Growth Factor Receptor
ESR1: Estrogen Receptor 1
PGR: Progesterone Receptor
TNBC: Triple Negative Breast Cancer
FDR: False Discovery Rate
LAR: Luminal Androgen Receptor Positive
QNBC: Quadruple Negative Breast Cancer.
