# Supplementary material for: Assessing the efficacy of androgen receptor and Sox10 as independent markers of the triple-negative breast cancer subtype by transcriptome profiling
Source: Oncotarget. 2018 Sep 7;9(70):33348–59. doi: 10.18632/oncotarget.26072 (PMC6161783; doi:10.18632/oncotarget.26072)
Supplement: Supplementary file 1 [file oncotarget-09-33348-s001.pdf]

Assessing the efficacy of androgen receptor and Sox10 as independent markers of the triple-negative breast cancer subtype by transcriptome profiling

SUPPLEMENTARY MATERIALS

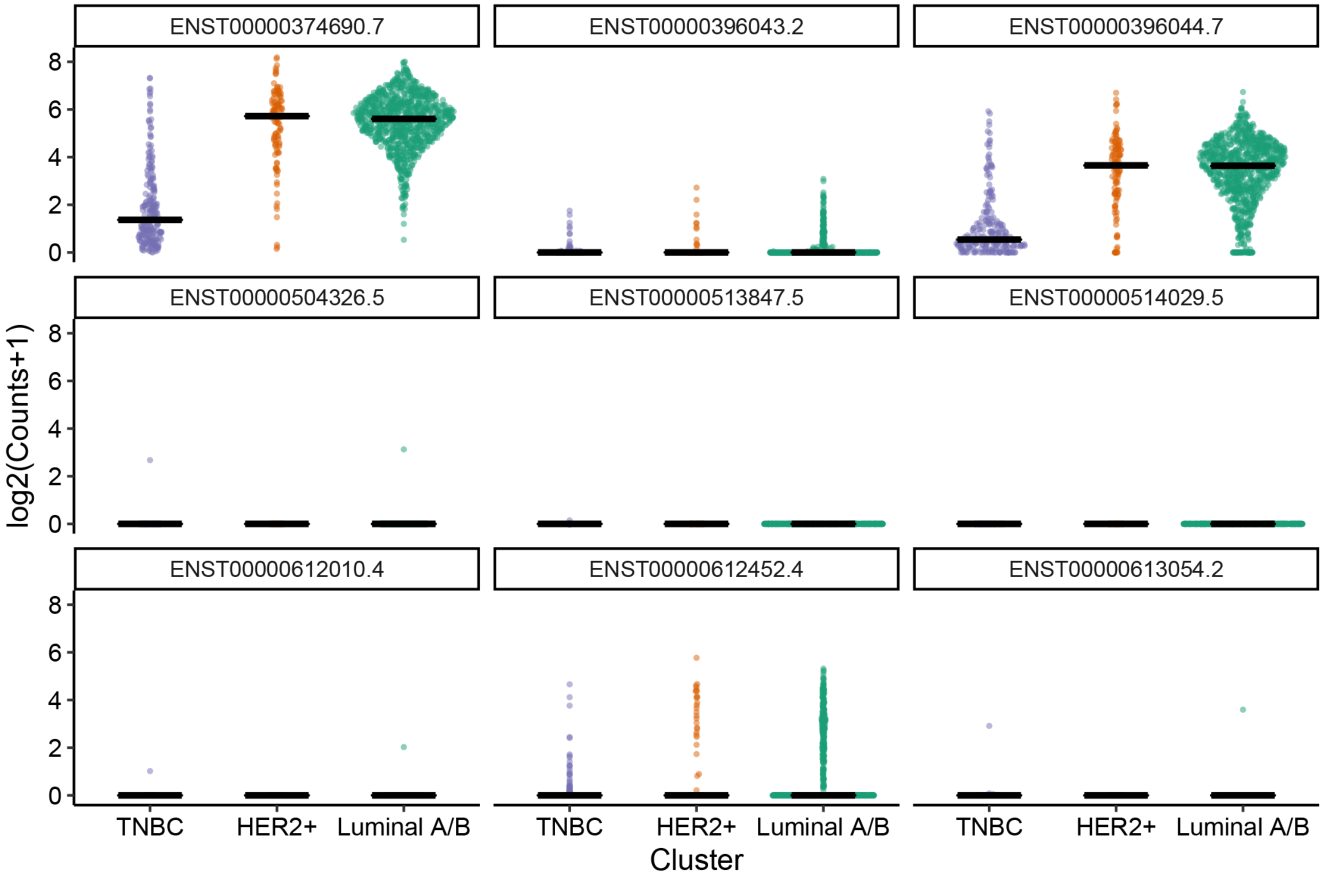

Supplementary Figure 1: Androgen Receptor isoforms do not correlate with different breast cancer subtypes. AR isoform expression data (log2 (counts+1)) was plotted for each patient sample from the TCGA dataset across the three molecular subtypes. Two predominant AR isoforms (ENST00000374690.7 and ENST00000396044.7) are expressed in human breast cancers. Both isoforms show a reduced level of expression within the TNBC subtype as compared to both the HER2+ and Luminal A/B subtypes.
